# What role does the GP play for emergency department utilizers? A qualitative exploration of respiratory patients’ perspectives in Berlin, Germany

**DOI:** 10.1186/s12875-020-01222-w

**Published:** 2020-07-30

**Authors:** Sarah Oslislo, Christoph Heintze, Martin Möckel, Liane Schenk, Felix Holzinger

**Affiliations:** 1 Institute of General Practice, Charité – Universitätsmedizin Berlin, corporate member of Freie Universität Berlin, Humboldt-Universität zu Berlin, and Berlin Institute of Health, Charitéplatz 1, 10117 Berlin, Germany; 2 Division of Emergency Medicine, Charité – Universitätsmedizin Berlin, corporate member of Freie Universität Berlin, Humboldt-Universität zu Berlin, and Berlin Institute of Health, Charitéplatz 1, 10117 Berlin, Germany; 3 Medical and Veterinary Sciences, James Cook University, The College of Public Health, 1 James Cook Dr, Townsville, Douglas, QLD 4814 Australia; 4 Institute of Medical Sociology and Rehabilitation Science, Charité – Universitätsmedizin Berlin, corporate member of Freie Universität Berlin, Humboldt-Universität zu Berlin, and Berlin Institute of Health, Charitéplatz 1, 10117 Berlin, Germany

**Keywords:** Emergency medicine, Primary health care, General practitioner, Qualitative research, Physician-patient relationship

## Abstract

**Background:**

While motives for emergency department (ED) self-referrals have been investigated in a number of studies, the relevance of general practitioner (GP) care for these patients has not been comprehensively evaluated. Respiratory symptoms constitute an important utilization trigger in both EDs and in primary care. In this qualitative study, we aimed to explore the role of GP care for patients visiting EDs as outpatients for respiratory complaints and the relevance of the relationship between patient and GP in the decision making process leading up to an ED visit.

**Methods:**

Qualitative descriptive study. Semi-structured, face-to-face interviews with a sample of 17 respiratory ED patients in Berlin, Germany. Interviews were recorded and transcribed verbatim. Qualitative content analysis was performed. The study was embedded into the EMACROSS (Emergency and Acute Care for Respiratory Diseases beyond Sectoral Separation) cohort of ED patients with respiratory symptoms, which is part of EMANet (Emergency and Acute Medicine Network for Health Care Research).

**Results:**

Three patterns of GP utilization could be differentiated: long-term regular consulters, sporadic consulters and patients without GP. In sporadic consulters and patients without GP, an ambivalent or even aversive view of GP care was prevalent, with lack of confidence in GPs’ competence and a deficit in trust as seemingly relevant influencing factors. Regardless of utilization or relationship type, patients frequently made contact with a GP before visiting an ED.

**Conclusions:**

With regard to respiratory symptoms, our qualitative data suggest a hypothesis of limited relevance of patients’ primary care utilization pattern and GP-patient relationship for ED consultation decisions.

## Background

EDs in many countries are faced with an increasing number of patients [1]; consultation reasons have been investigated in studies from a variety of international settings [2]. Respiratory complaints constitute an important utilization trigger in this context [3, 4] and also one of the principal consultation reasons in GP practices [5, 6]. ED utilization of patients with less urgent complaints that could have been potentially managed in primary care are considered to contribute to ED crowding, ultimately increasing the potential for adverse outcomes for all patients in the ED [7, 8].

Health-related anxiety and the surmised availability of advanced diagnostics in the hospital setting have been discussed as important factors in the complex decision making process leading up to ED utilization, as well as a lack of connection to continuous primary care [3, 9, 10]. The issue of interactions between GP care and ED utilization however, while briefly raised in a number of publications, has not been comprehensively investigated [11–13]. Why patients who are embedded in a functioning continuous care relationship with a GP might still frequently consult EDs and whether these patients’ utilization differs from patients without regular GP attachment remains unresolved. The role of the GP in individual patients’ – acute and chronic – medical care might be very different, depending on factors ranging from doctor-patient relationship to the GP’s function in the respective health care system [14, 15]. GPs constitute a central pillar of primary care in many health systems [15], but the demarcation of primary care tasks and the role of hospital EDs in acute medicine is frequently not clearly defined. Choice of point of care is often left to the patient in absence of explicit rules or steering mechanisms.

In Germany, almost 90% of the statutory health insurance members report to have a regular GP [16], but ED consultations are nevertheless abundant, including a considerable proportion of cases treated as outpatients and not admitted to hospital after the diagnostic process [17]. Patients in Germany are not required to register with a specific GP practice and there is no such thing as a central medical file. Thus, considering a particular GP as one’s “fixed” or “regular” provider is a matter of the individual patient’s personal judgment. The German health care system does neither include a gatekeeping procedure: while patients might be referred to the ED by a GP or specialist practice, they also can go there at their own discretion. Many settings are comparable in this regard [18, 19]. There are also no penalty charges for consulting an ED without being referred.

In this qualitative study, we explored the relationship and interactions between GP care and ED outpatient visits from the patient perspective. The study was embedded in a cohort study investigating ED care for patients with respiratory symptoms. Our research questions were: What role does the GP play in these ED patients’ usual health care? Is the relationship with the respective GP relevant in the decision making process ultimately resulting in the ED consultation?

## Methods

Study design and results are reported in this article in line with the COREQ criteria [20].

### Study background

This qualitative descriptive study is a module of the mixed-methods research project EMACROSS which investigates the characteristics, motives and health care utilization of patients with respiratory symptoms visiting EDs in a network of eight hospitals in the central district of Berlin, Germany. It is a subproject of the health care research network EMANet [21].

### Sampling of participants

The study features an embedded design; participants were recruited as a sub-sample of the prospective EMACROSS cohort. Inclusion criteria for EMACROSS were: adults (no maximum age), all gender, and treatment in a participating ED due to respiratory symptoms. Patients were excluded if unable to give informed consent [22]. For this qualitative study, only patients not admitted to hospital after the ED consultation were potentially eligible. All such outpatients recruited for the quantitative cohort and completing the baseline survey were asked at this point whether they would be prepared to take part in an additional qualitative interview, scheduled separately. Interview partners were sampled from the resulting pool of potential responders by a strategy comprising some basic elements of purposive sampling [23]. We aimed for contrasting basic patient characteristics regarding age, gender, and GP status (does the patient have a regular GP or not?). The overall cohort comprised ~ 10% of patients without a GP, but it seemed prudent to include a proportionally larger number of such patients into the qualitative sample due to our research questions. Medical characteristics (e.g. diagnoses) were not used as a sampling basis, as this information was not comprehensively available from the cohort dataset at the time of participant selection. Thus, it is certainly arguable whether the balancing of basic demographics and intentional over-representation of patients without a GP is sufficient for considering participant sampling purposive rather than convenience. Scheduling of interviews was permitted for a maximum of two weeks after the index ED visit to limit recall bias.

### Data collection

Based on the literature [10, 14, 24, 25] and theoretical deliberations, a semi-structured interview guide was developed. We aimed for a great degree of openness to allow patients to relate their views and experiences freely. The guide was repeatedly adapted after discussion within the research team and in an interdisciplinary qualitative research group including scientists not directly involved in the study. After a pretest and again after a first set of interviews, questions were revised [26]. The guide was finalized after the fourth interview (see Table 1 for an excerpt, and Additional file 1 for the full set of questions). For an excerpt, and Additional file 1 for the full set of questions). It was used flexibly to permit a natural conversation flow [27]: we did not rigidly adhere to the order of questions nor did we pose a question if the topic had already been comprehensively covered. In interviews with patients without a GP, phrasing was adapted to match the situation and nevertheless gain insight into the interviewees’ view of GP care.

**Table 1:** Questions from the interview guide (excerpt)

• To what extent was your GP / a GP involved in the decision making process that led to your ED visit?	
• Did you contact your GP / a GP before visiting the ED? Why did you choose to do so?	
• What role does GP care play in your health care? Why?	
• How would you describe your relationship to your GP? Why?	
• Patients without GP: How would you describe your past experiences and relationships with GPs? Why?	

During recruitment, we contacted a total of 24 patients from the EMACROSS cohort. Seven refused to participate; the most frequent reasons stated were lack of time for an interview and the perceived severity of their health condition. Between August 2017 and May 2018, 17 patients were interviewed face-to-face at their home, workplace or at our university by an experienced health scientist (SO), interviewees were informed about her background and the reasons for conducting the study. Participants gave written informed consent a priori. At all but one interview, only the interviewer and the participant were present, one interviewee had requested the presence of his wife. Interviews had a mean duration of 25 min and were audio-recorded. Field notes were taken parallel to the interviews. Verbatim transcription and preliminarily coding were conducted parallel to the interview phase. Transcripts were pseudonymized. Data collection was concluded when interviews appeared not to yield any additional findings based on the provisional coding framework, indicating content saturation [28]. Transcripts were not returned to the participants for comments or correction. Repeat interviews were not conducted. Interviews were conducted in German language; data was translated into English by the authors for the purpose of this publication.

### Data analysis

Qualitative content analysis was performed [29] (SO, health scientist, researcher and doctoral candidate in the EMACROSS project, Institute of General Practice). Content was first structured according to a deductively developed category system and coding guideline, based on the literature and theoretical considerations. A subset of the material was coded; additional categories were developed inductively based on themes raised in the interviews. The coding guideline was subsequently revised. Part of the material was coded by a second researcher independently (FH, general practitioner and senior researcher, Institute of General Practice), results were discussed and unclear or indistinct categories revised again. The guideline was also repeatedly discussed with external qualitative researchers. Distinct definitions and corresponding anchor examples from the interviews were appropriated to thematic categories and subcategories to obtain a clear assignment of quotations. After finalization of the category system, the remaining material was coded; initially analysed content was re-coded to assess fit of the newly formed categories. The combined deductive-inductive analysis procedure enables both consideration of the theoretical framework and identification of new and additional themes from the interviews [30, 31]. MAXQDA 2018 software was used to support the management of the coding process. Participants were not asked to provide feedback on findings.

## Results

### Sample characteristics

We conducted a total of 17 interviews. The qualitative sample essentially corresponds to the EMACROSS cohort in regard to basic demographics (interim analysis: gender f/m 46/54%; mean age 53; unpublished data). Due to the sampling strategy, the proportion of patients without regular GP attachment was greater than in the source cohort. Table 2 gives an overview.

**Table 2:** Characteristics of study participants, *n* = 17

Patient characteristic	***n*** (%)
**Gender**	
Female	9 (52.9)
Male	8 (47.1)
**Age group, years**	
20–39	5 (29.4)
40–59	6 (35.3)
≥ 60	6 (35.3)
*Mean*	*50.9*
*Median*	*49.5*
**GP status**	
Yes	13 (76.5)
No	4 (23.5)
Morbidity*	
Chronic obstructive pulmonary disease (COPD)	8 (47.1)
Asthma	2 (11.8)
Acute respiratory tract infection (RTI), e.g. bronchitis, pneumonia	4 (29.4)
Subjective dyspnoea, exclusion of serious illness (like e.g. pulmonary embolism)	3 (17.6)
ED consultation*	
Out-of-hours	6 (35.3)
Referred by physician**	7 (41.2)
Triage category*^†^	
2 very urgent	1 (5.9)
3 urgent	8 (47.1)
4 standard	6 (35.3)
5 non urgent	2 (11.8)
Subjective urgency*	
1 must be seen immediately	5 (29.4)
2 must be seen as soon as possible	7 (41.2)
3 must be seen today	3 (17.7)
4 less urgent	2 (11.8)
Previous ED visit in past 6 months*^‡^	7 (41.2)
GP visit in past 6 months*^‡^	13 (76.5)

*Determined post hoc from quantitative cohort dataset, not a sampling criterion

**Six of these patients: ED consultation during practice office hours

^†^Manchester triage system, categories 1–5. None of the participants had been triaged as category 1 (immediate)

^‡^At least one visit of respective institution/health care provider

### The role of GP care

After categorizing interview content relating to the role of respiratory patients’ GP care, three distinct patterns of GP utilization emerged: *long-term regular consulters*, *sporadic consulters* and *patients without GP.* Characteristics delineating these types of primary care utilization are summarized in Table 3. In the following sections, this typology will be depicted in detail on the basis of exemplary quotations.

**Table 3:** Role of GP care: patient types and characteristics

Long-term regular consulters	• GP central as first contact person • Important role in chronic disease care • GP as supporter and health advisor
Sporadic consulters	• Occasional GP visits, heterogeneous utilization • Factors: skepticism concerning competence, lack of confidence, past negative experiences
Patients without GP	• GP has no important role • Prevalent mistrust in regard to GPs’ skills and knowledge • Limited demand due to good health condition

#### Long-term regular consulters

This group comprised eight interviewees who reported to generally regard the GP as the first point of contact for health concerns.

‘When I'm sick, I always go to the GP.’ (P10, male, in his 20s)

Almost all *long-term regular consulters* reported to have contacted their GP before visiting the ED, most were referred there. One patient related futile efforts to reach his GP on the weekend, and another interviewee decided to go to the ED directly without consulting the GP due to a high subjective urgency. Patients stressed the central importance of the GP in their continuous medical care, for example in monitoring and treating chronic disease. The GP was depicted as a crucial advisor and supporter in all medical matters.

‘A large role [...] because from my point of view, he can answer all my questions if I have problems with my health.’ (P6, female, in her 50s)

‘Actually a very large one, because […] he principally determines what needs to be done to improve my health.’ (P10, male, in his 20s)

#### Sporadic consulters

These patients (five interviewees) reported to have a GP and also related occasional visits, but utilization of GP care was described as heterogeneous, depending on the situation. Compared to the *long-term regular consulters* group, the relevance of the GP as the first contact for health-related matters was depicted as much less pronounced. Nevertheless, all interviewees in this group reported to have consulted – or tried to consult – a GP prior to their ED visit.

‘[...] I wanted to see a GP on Monday. But there was no one and no replacement.’ (P15, male, in his 40s)

The comparable lack of relevance of GP care in *sporadic consulters* was attributed to a diverse set of reasons. Some interviewees described past experiences of dissatisfaction with GPs, like not being issued with a prescription of antibiotics when they desired so. Additionally, a general but mostly unspecified lack of confidence was frequently expressed, as well as scepticism regarding the GP’s comprehensive view of their health situation.

‘And I think, the GP [...] does not have such an overview. I don’t really trust him anyway.’ (P8, female, in her 60s) The capacities of GP care were seen as limited by some interviewees, and time constraints were also mentioned critically.

'When I go there, he has little time […]. It is not great.’ (P3, male, in his 60s)

Interviewees frequently expressed scepticism in regard to GPs competence due to their generalist orientation, and some clearly stated preference of specialist medical care. Participants voicing such attributed to specialists greater competence and superior overview, especially in cases of chronic disease.

‘He is so general, you could say, just like myself. [...] I ask him something, then he takes a big book and starts reading. This is something I could do myself […], if I have questions.’ (P5, female, in her 40s)

‘Compared to my lung specialist he is a zero, the GP.’ (P3, male, in his 60s)

Some interviewees described a change over time: the GP used to play an important role (comparable to *long term regular consulters*), but this has diminished as a result of negative experiences.

‘So he has played a very big role for a while. But [...] I decided not to go there anymore. Because I ended up in hospital every time.’ (P11, female, in her 50s)

#### Patients without GP

The four patients in this group reported to *have no GP*, meaning no attachment to or repeated visit of a specific GP practice. However, the interviews showed that this does not exclude instances of eventual situational GP utilization: three of the four patients in this group tried to contact a GP practice before visiting the ED, and the results of these contacts do not substantially differ from the other patient groups: some were referred to the ED, while unsuccessful attempts of contact eventually triggered ED self-referrals in other instances.

‘In the morning I tried to call the doctor, this big GP practice. And I couldn’t get any contact.’ (P13, female, in her 60s)

Altogether, patients in the *no GP* group attributed their habitual non-utilization of GP care to two main reasons: mistrust in regard to GPs’ competence – subjectively confirmed by personal experiences – and an absence of any real need for regular medical care due to good health.

‘I wanted a diagnosis. [...] He just couldn't determine what I had [...]. And that disturbed me so much [...]. [...] So neither a smear nor anything. I would have wished that he […] actually would investigate.’ (P14, female, in her 30s)

‘No GP, because I [...] am in fact rarely ill. That's why I don't actually utilize these physicians.’ (P14, female, in her 30s)

### Relationship to GPs

The above-noted primary care utilizer typology derived from the interviews was based on respiratory patients’ depiction of their attachment – or lack of such – to GP practices and their related primary care consultation patterns. In this context, the individual doctor-patient relationship described by the patients emerged as an important influencing factor, which is already discernible to some degree in the quotations related in the previous section. Interviewees’ respective statements allowed for a categorization into three classes: *positive and supportive relationship*, *ambivalent relationship* and *aversive relationship* (see Table 4).

**Table 4:** Characteristics of GP-patient-relationship: categories

Positive and supportive	• Strong emphasis on favorable aspects of doctor-patient-relationship • Interviewees relate positive experiences
Ambivalent	• Relationship has both positive and negative facets • Mixed experiences
Aversive	• Relationship is primarily experienced and depicted as negative

#### Positive and supportive relationship

Patients who predominantly depicted the connection to their GP as positive and beneficial stressed three main aspects: friendly interaction and sympathy, trust in the physician’s abilities, and intimate knowledge of the patient’s situation due to long-term mutual acquaintance.

‘I would say very friendly. [...] I mean, I have known him for over twenty years.’ (P12, male, in his 50s)

‘[…] I have noticed that it is better to have a doctor who knows a bit about the story.’ (P16, female, in her 30s)

‘Because I feel comfortable there. I know this doctor and he is competent, he is nice. And there is a degree of trust, too.’ (P10, male, in his 20s)

All eight *long-term regular consulters* as well as one patient from the *sporadic consulters* group related an overall positive and supportive relationship.

#### Ambivalent relationship

Two interviewees were categorized in this group, based on mixed statements in regard to their individual doctor-patient relationship. While friendliness and efforts made by the GP were remarked as positive, the frequently impersonal nature of doctor-patient interaction was criticized. This lack of connection ultimately contributed to the decision to favor the ED in one case.

‘He is nice, […] and he makes an effort.’ (P11, female, in her 50s)

‘[…] but of course, it is very impersonal. [...] another reason why I went to the ED, because my trust in the ED was simply greater than in a GP whom I don’t even really know.’ (P15, male, in his 40s)

The same patient related dissatisfaction with the limited opening hours and availability of the practice he sporadically frequents.

‘And the service level is so bad: Monday in the afternoon 13:00 to 15:00, and Tuesday morning 9:00 to 12:00, and Wednesday not at all […] this is super unprofessional [...] seems more like a hobby practice to me.’ (P15, male, in his 40s)

Another patient attributed the *ambivalent relationship* to past disappointments resulting in a loss of trust, namely a number of instances where she had the impression of having been prescribed antibiotics too late by the GP, finally resulting in a necessity for inpatient care.

#### Aversive relationship

Two patients from the *sporadic consulters* group leaned towards a predominantly negative assessment of their GP-patient relationship. Main areas of criticism were the impersonal character of the care received, time constraints, as well as perceived deficits in GPs’ knowledge about the patients’ situations, resulting in a general lack of trust. Corresponding themes had been raised in the *ambivalent* group, but were not balanced by a concomitant portrayal of positive aspects here.

‘Now, I don't think he knows me very well, […] I don’t really trust him. (P8, female, in her 60s)

As already outlined, patients *without a GP* nevertheless related views and past experiences concerning GP care. As in the statements of patients consulting their GP at least *sporadically*, but describing and overall *aversive relationship,* lack of trust was strongly thematized. As to trust loss, patients reported episodes of feeling insufficiently investigated and treated by GPs in the past.

‘[...] we were a total mismatch. I had to point out that he should please investigate this [...]. […] I had to worm everything out of him […]. And I didn’t like that. […] I just didn't feel well looked after anymore.’ (P14, female, in her 30s)

‘Trust [...], that’s what I miss here.’ (P13, female, in her 60s)

‘I know, there was this epidemic recently and they just had this ‘one size fits all’-approach. And in my case, it was worse, and it annoyed me so much (…) this was overlooked. ’ (P14, female, in her 30s)

‘[…] always just listened to my lung and wanted to give me stronger medication for obstruction, but never a blood analysis.’ (P13, female, in her 60s)

## Discussion

### Summary of findings

In our sample of respiratory ED patients, three general patterns of GP utilization could be differentiated: *long-term regular consulters*, *sporadic consulters* and *patients without GP*. While the GP plays a central role and prevailingly constitutes the first point of contact for *long-term regular consulters*, interviewees characterised as *sporadic consulters* reported an altogether heterogeneous utilization behaviour. Lack of confidence in GPs’ professional capacities with a resulting belief in the superiority of specialist care seems to play a role in this context, as well as negative experiences with GPs. The group of *patients without a GP* predominantly attributed GP-based primary care a low relevance for their individual care context. Besides limited necessity due to a good health status, general mistrust in regard to GPs’ competence was prominently voiced, corresponding to the issues raised in the *sporadic consulters* group. As personal trust in the GP’s abilities as well as knowledge of the patient’s history seemed to constitute influencing factors for GP utilization, the individual doctor-patient relationship is of particular interest. In this regard, a basically *aversive* view of GP care was discernible in a number of patients with a *sporadic consultation* pattern. Negative experiences seemed to be potentially contributive to a deterioration of the respective patient’s view of GP care. Perceived shortcomings encompassed patients’ impression of the respective GP not knowing or remembering a lot about them, a felt lack of time in consultations or unmet expectations concerning diagnostics or prescriptions. Speculatively, this could result in *sporadic consulters* with an *aversive* view to cease utilization of GP care.

Overall, we gained the impression of a limited influence of primary care consultation pattern, general view of GP care and personal GP-patient relationship on respiratory patients’ behaviour in acute situations. A large majority of our interviewees reported to have consulted a GP before resorting to hospital, or at least endeavoured to do so. This also applies to patients in the *sporadic consulters* group, and even *patients without a GP* preferred this approach to outright ED self-referrals. Interestingly, this was likewise related by patients with a generally GP-*aversive* attitude.

### Strengths and limitations

The question of the role of both a patient’s GP care utilization and her or his relationship with a GP in the context of ED consultations has not been studied extensively, thus our study adds a new perspective to the discussion of ED utilization reasons. The qualitative research process allowed for a detailed exploration of patients’ views and underlying experiences. A number of limitations apply. Concerning transferability of findings, it must be noted that – due to the focus of the study it was embedded in – the sample consisted of patients with respiratory symptoms as trigger of the index ED visit. Views of patients with other morbidity characteristics might differ – this important issue will be further discussed in the following “results in context” section. Additionally, transferability to other settings may be complicated in case of divergent health system characteristics, e.g. in countries with strong primary care gatekeeping elements or compulsory attachment to GP practices. It should also be borne in mind that any interview study’s trustworthiness relies on how participants relate their considerations, decisions and actions, and social desirability bias might be an issue of concern [32]. In light of the public debate about “inappropriate” ED utilization, even if assured of pseudonymized analysis, patients might claim to have consulted or tried to consult a GP before self-referring there, retrospectively feeling that this would probably have been expected of them. In regard to categorization of patients and delineation of subgroups based on interview content, the inherently subjective nature of text interpretation poses a degree of difficulty. Some classifications could be considered arbitrary to a degree (e.g. *aversive* vs. *ambivalent*). However, we strived to minimize this by use of independent coding and external review in methods workshops [33]. Constant reflection of the research process inside and outside our team also was an important measure for delimiting unwitting influence of researchers’ prior theoretical assumptions and convictions [34]. With regard to the typologies of the GPs’ role in patients’ health care utilization and of GP-patient relationships, we must add that this did only crystallize from earlier codes later in the analysis process, and after termination of interviews. Thus, interpretative caution is warranted as nonattainment of saturation in the subgroups is quite possible.

Concerning the deliberations on morbidity and urgency in our ensuing discussion, we would like to stress that the numbers (e.g. proportions of diagnoses) stated do certainly not reflect any kind of prevalence and can self-evidently not claim representativeness in a statistical sense. The small sample and qualitative methodology prohibit any additional quantifications or inferences. It must also be borne in mind in this context that the information was derived post hoc from the cohort dataset and did not constitute a factor in the sampling of participants. We still decided to discuss these aspects because pattern recognition constitutes an important aspect of qualitative data analysis [35], and ignoring their potential influence would have constituted a substantial limitation in our view.

### Results in context

In our sample of patients with respiratory complaints, we gained the impression that neither the general role of the GP in patients’ health care nor the individual doctor-patient relationship seemed to constitute a central factor in the decision making leading up to an ED consultation.

#### Relevance of primary care and connection with ED utilization

A considerable number of studies have tried to shed a light on the question of why some patients have high ED utilization while others do not, and whether primary care plays a role here. However, evidence is heterogeneous and even contradictory. Having a GP and a continuous doctor-patient relationship has been shown as potentially associated with lower ED use in some studies [36, 37], especially in the elderly or in special situations like end-of-life care [9, 38, 39]. A positive effect of a decline in ED visits has also been reported for improved primary care coordination in difficult social contexts [40]. Patients without a GP were characterized as potentially prone to higher ED utilization [41, 42], and also to a higher proportion of non-urgent consultations [37]. In contrast, others have concluded that ED utilization is mostly driven by patients’ health conditions and care needs rather than their primary care situation [43]. A number of studies come to the conclusion that high utilization of GP services in fact seems to go along with a concomitant increased ED consultation rate, and that these patterns are not sufficiently explained by patients’ morbidity [44–46]. In a study from Taiwan investigating the relationship between utilization patterns of different health services, ED patients could be characterized as *low health care users*, *hospital fans*, *primary care favorers* or *high health care users*, and extensive ED usage was heavily associated with high consultation in other sectors [45]. We could not identify any study attempting a similar typology of utilization in regard to GP care.

The at first glance surprising circumstance of most of the ED patients in our study reporting a consultation – or at least an attempt – with a GP prior to their ED visit is consistent with the results of Benger and Jones [47], who investigated ED consulters in Bristol, UK. This fits in with the studies describing ED utilizers’ parallel high use of other health care services [44–46].

#### GP-patient relationship as an influencing factor

Concerning the individual doctor-patient relationship, personal trust in – or doubt about – the GPs’ professional competence transpired as a central influencing factor for both utilization behavior and general attitude towards GP care in our study, and this was depicted as heavily influenced by past patient experiences. In this respect, a number of international studies have emphasized trust as a pivotal component of the doctor-patient relationship [14, 48, 49]. Lings et al. [48] stressed that this concept encompasses trust in the personal integrity as well as in the medical competence of the physician. Development of trust also is related to the continuity of the doctor-patient relationship [50]. The notion of loyalty, which is mainly based on trust [51], is important in this context: Roberge et al. [25] illustrated that a sustained partnership, once established, is in no way a permanent given, but rather is periodically reconsidered by patients in light of their evaluation of their physicians’ medical competence and personal skills. This corresponds to the changing utilizations patterns reported by some of our interviewees: a switch from *regular consulting* to *sporadic consulting* or even “giving up” GP care is frequently triggered by unsettling care experiences. The same can be said for a change of general attitude towards GP care: *aversive* views frequently tend to be rooted in negative experiences. On the other hand, a patient’s impression of the physician investing time was depicted as an important beneficial factor for a positive and sustained doctor-patient relationship, which is in line with the results of other studies [14, 48, 52, 53]. Meeting the patients’ expectations and needs has also been stressed as instrumental [14, 54], and this was likewise confirmed by our interviews. Naturally, these aspects are of greater importance for patients in regular need of medical care, e.g. because of chronic illness, whereas generally healthy people might not see any necessity to attach themselves to a GP. This corresponds well to the notion of vulnerability procreating the need for attachment raised by Frederiksen et al. [55].

#### Considerations of morbidity and urgency

When discussing our results in the context of international literature on interactions between GP utilization, GP-patient relationship and ED consultations, it must however be borne in mind that our interview sample consisted of patients with a selected spectrum of symptoms. To our knowledge, there is no preceding study which has explored a similar research question in a population directly comparable in terms of morbidity. As already mentioned in the description of limitations, this gives rise to some caution regarding transferability of findings. Respiratory complaints constitute a very frequent consultation reason in EDs [3, 4], so we do not talk about an extraordinary or rare patient subgroup. Nevertheless, respiratory complaints have some particular characteristics that may impact consultation behaviour: subjective perception of dyspnoea, which is a frequent respiratory symptom, and the associated feeling of being threatened, may trigger ED visits [56].

Among respiratory ED patients, there also is a high prevalence of chronic pulmonary conditions like asthma and COPD [57], which is likewise shown by our quantitative cohort dataset (> 40%). In our qualitative sample, 10 of the 17 patients (59%) had underlying chronic respiratory conditions. Considering the utilization typology derived from our interview material, patients *without a GP* show the lowest proportion of chronic pulmonary conditions (25%), compared to *sporadic* (80%) or *long-term regular consulters* (63%). This reflects in the general good health stated by some of the interviewees as a reason for not relying on GP care.

Chronic pulmonary morbidity also has considerable impact on healthcare utilization: a higher symptomatic burden is associated with increased utilization in all sectors including primary care [58]. In patients with chronic pulmonary diseases like COPD, inadequate disease control with subsequent exacerbations constitutes a frequent problem in primary as well as specialist pulmonary care [59]. This could contribute to patients feeling dissatisfied with their treating physicians, which may mirror in our interviewees’ depiction of their attitude towards GP care. Then again, the few studies depicting satisfaction of chronic pulmonary patients in primary care have not indicated discontent among this patient group as a distinctly prevalent problem [60, 61]. Concerning GP-patient relationship, the proportion of patients with chronic pulmonary morbidity was not strikingly discrepant in GP-supportive and GP-aversive patients in our sample (67 and 75%). Considering any kind of disease, only four patients in our sample did not report a chronic condition (pulmonary or other), and two of these “healthier” interviewees nevertheless reported a *long-term regular consulting* pattern in regard to GP care.

Altogether, no clear picture emerges from these considerations of morbidity, and their relative importance in comparison with the underlying interpersonal factors – like trust in the physician and the feeling of being well cared for – that seemed to influence our interviewees’ utilization decisions remains unclear to a degree.

Another point which should be looked at when talking about ED consultations and their underlying reasons is subjective urgency: patients with a usually good integration into primary care structures could nevertheless have decided that the ED was the appropriate care level, because they may have considered their acute medical situation as urgent and dangerous. In our sample, subjective urgency was considerable (see Table 1**,** derived from respective item in the quantitative survey). In such constellations, patients’ usually sound relationship to a GP could potentially be of little consequence [62].

## Conclusions

In our sample of ED patients with respiratory symptoms, we could identify patterns of both primary care utilization and patients’ relationship with GP care. Our qualitative data suggest a hypothesis of limited impact of both attributes on patients’ decision making in regard to ED visits. Interviews in fact suggest a high potential readiness to be treated by a GP in cases of acute respiratory complaints, even in patients not continuously attached to a practice. Whether this applies to patient groups with other morbidity spectrums should be evaluated in future studies. The validity of the notion of achieving a reduction in ED consultations by strengthening primary care attachment and care continuity likewise has to remain subject to further scientific investigation, as respective intervention studies are scarce. In this context, it is neither sure whether patients without a GP would be willing to attach themselves to a practice (even if provided with appointments, thus eliminating conceivable access barriers) nor whether such would result in fewer ED visits. Evaluating both acceptability and effectiveness would require a mixed methods approach, in which qualitative explorations would constitute an important part.

## Supplementary information

**Additional file 1.** Interview guide questions. Full set of questions from the interview guide

## Data Availability

The data in this paper is based on 17 audio-recorded interviews with patients and the respective transcripts. The data supporting the findings of this study is represented by the translated quotes in the results section of this article. However, the data generated and analysed during the study (audio files and transcripts) is not publicly available, as it may still contain information that could potentially compromise the privacy of our participants.

## Abbreviations

GP: General practitioner
ED: Emergency department
