# Supplementary material for: What role does the GP play for emergency department utilizers? A qualitative exploration of respiratory patients’ perspectives in Berlin, Germany
Source: BMC Fam Pract. 2020 Jul 30;21:154. doi: 10.1186/s12875-020-01222-w (PMC7393893; doi:10.1186/s12875-020-01222-w)
Supplement: Supplementary file 1 — Additional file 1. Interview guide questions. Full set of questions from the interview guide [file 12875_2020_1222_MOESM1_ESM.pdf]

Interview guide questions

Context and initiation of ED visit

- Could you please tell me how your ED visit came about?
- How did you decide to go to the ED?
- To what extent was your GP / a GP involved in the decision making process that led to your ED visit?
- How did you choose the ED you visited?
- What expectations did you have concerning the ED visit?
- Did you contact your GP / a GP before visiting the ED? Why did you choose to do so?

ED visit

- How did you experience your ED visit?
- Could there have been alternative care options for your situation?
- Would you go to the ED again with similar complaints? Why?
- Has anything changed in your life or health since the ED visit?
- What did you do after your visit to the ED? Why?
- Have you been in an ED on other occasions in recent years? If so, are there any experiences you remember specifically in this context? Why?
- For what kind of health problems should one go to an ED or rather to a doctor's office?
- In retrospect, would you say that you were an emergency? Why?

Usual health care, GP care

- Where do you usually go if you are ill?
- In your opinion, what are the functions of a GP?
- What role does GP care play in your health care? Why?
- How would you describe your relationship to your GP? Why?
- Patients without GP: How would you describe your past experiences and relationships with GPs? Why?
- Are there other physicians, therapist etc. you visit regularly? If so, on what occasions?

Additional questions

- Do you have any suggestions or ideas for improvement of ED care?
- Is there anything else you would like to tell us that has not been addressed so far?
